# The Important Role of Phosphatidylserine, ADAM17, TNF-Alpha, and Soluble MER on Efferocytosis Activity in Central Obesity

**DOI:** 10.1155/2024/1424404

**Published:** 2024-03-20

**Authors:** Chandra Agung Purnama, Anna Meiliana, Melisa Intan Barliana, Keri Lestari, Andi Wijaya

**Affiliations:** ^1^Department of Pharmacology and Clinical Pharmacy, Faculty of Pharmacy, Universitas Padjadjaran, Jl. Ir. Soekarno Km 21, Jatinangor 45363, Indonesia; ^2^Prodia Clinical Laboratory, Jl. Kramat Raya 150, Jakarta 10430, Indonesia; ^3^Prodia Education and Research Institute, Jl. Kramat Raya 150, Jakarta 10430, Indonesia; ^4^Department of Biological Pharmacy, Faculty of Pharmacy, Universitas Padjadjaran, Jl. Ir. Soekarno Km 21, Jatinangor 45363, Indonesia; ^5^Center of Excellence of Pharmaceutical Care Innovation, Universitas Padjadjaran, Jl. Ir. Soekarno Km 21, Jatinangor 45363, Indonesia

## Abstract

**Background:**

Obesity is expected to hinder efferocytosis due to ADAM17-mediated cleavage of the MER tyrosine kinase receptor, producing soluble MER (sMER) that disrupts MERTK binding to cell death markers. However, the intracellular efferocytosis pathway in central obesity remains elusive, particularly the role of low-grade chronic inflammation in its initiation and identification of binding signals that disrupt efferocytosis.

**Objective:**

We investigate the efferocytosis signaling pathway in men with central obesity and its relationship with inflammation, cell death, and related processes.

**Methods:**

A cross-sectional study was conducted, and clinical data and blood samples were collected from 56 men with central obesity (obese group) and 29 nonobese individuals (control group). Clinical evaluations and predefined biochemical screening tests were performed. The efferocytosis signaling pathway was investigated by measuring phosphatidylserine (PS), ADAM17, TNF-alpha (TNF-*α*), and sMER.

**Results:**

Metabolic syndrome was detected in more than half of the participants in the obese group according to the predefined tests. Mean levels of PS, TNF-*α*, and sMER were higher in the obese group but not significantly different from those of the control group. Further analysis based on waist circumference (WC) ranges in the obese group revealed a significant increase in PS and sMER levels between the control group and the obese group with WC greater than 120 cm. ADAM17 levels were significantly higher in the obese group than in the control group. PS was positively correlated with WC and ADAM17. ADAM17 was positively correlated with TNF-*α* and sMER, indicating impaired efferocytosis.

**Conclusions:**

Central obesity appeared to cause a disturbance in efferocytosis that began with cell damage and death, along with an enlargement of the WC and an ongoing inflammatory response. Efferocytosis was disrupted by proinflammatory cytokine regulators, which induced the production of sMER and interfered with the efferocytosis process.

## 1. Introduction

Obesity has become a global epidemic in the past decade and has grown rapidly in developing countries, including Indonesia [1]. To put it simply, obesity is primarily caused by excessive calorie intake. Fat accumulation in the visceral area is known to lead to metabolic abnormalities, referred to as “central obesity” by the World Health Organization (WHO) for the Asia-Pacific region, and is defined as a waist circumference (WC) greater than 90 cm in males [2, 3].

During central obesity, hypertrophy of the adipose tissue triggers the unbalanced secretion of adipokines and increases the production of free fatty acids, leading to metabolic syndrome and modulates inflammatory reactions, glucose, and lipid metabolism [4]. Increased cell death during central obesity leads to increased macrophage infiltration, which creates a crown-like structure (CLS) that surrounds the dead adipocytes [5]. Tumor necrosis factor alpha (TNF-*α*) cytokines are mainly produced by adipose tissue macrophages, which are proinflammatory [6]. TNF-*α* converting enzyme, also known as a disintegrin and metalloproteinase 17 (ADAM17), regulates the expression of these cytokines [7] and is activated by the transfer of phosphatidylserine (PS) to the outer membrane of adipocytes during cell injury and death [8]. The increased effect of ADAM17 in central obesity is related to a decrease in the expression of the tissue inhibitor metalloproteinase 3 (TIMP3) [9].

Adipocyte cell death is apoptotic and is removed by phagocytes, particularly macrophages, as part of the cell remodeling process. Rapid and efficient clearance of apoptotic cells by macrophages is critical to maintain tissue homeostasis and prevent secondary necrosis, which increases inflammation [10, 11]. This process of clearing apoptotic cells is called efferocytosis. The efferocytosis process is divided into three stages: recognition, binding, and cell death degradation [12, 13]. Various markers of each stage have been identified. However, these identified markers are limited to *in vivo* testing. One of these markers is the MER proto-oncogene tyrosine kinase (MERTK) receptor, which plays a pivotal role in the binding process. This receptor is located on the surface of macrophages and binds to bridge molecules produced by apoptotic cells, such as protein S and growth arrest-specific protein 6 (Gas6), which have a high affinity for PS [14, 15]. Efferocytosis impairment is predicted due to interference with the MERTK receptor. The research by Suresh Babu et al. showed that efferocytosis was altered in macrophages of obese and diabetic mice [16]. This impairment was caused by the shedding of MERTK by ADAM17, which functions as the main protease [16]. The proteolytic cleavage of MERTK produced soluble MERTK (sMER) [17], which interacts with Gas6 and protein S, thus disrupting its binding with the MERTK receptor on macrophage membranes. This decreases the intracellular downstream signaling that modulates efferocytosis and the resolution of inflammation [16, 18]. However, further research is needed to investigate this intracellular efferocytosis pathway in humans with central obesity.

By recognizing the global urgency of understanding obesity-related processes, our study emphasizes the importance of noninvasive methods, such as blood tests, for ethical and scalable research. This method helps us study a larger group of people, ensuring more reliable findings. In this study, our aim was to understand efferocytosis signaling in central obesity, especially how low-grade chronic inflammation triggers the process and to identify binding signals that activate efferocytosis. Markers important in obesity and efferocytosis (e.g., PS, ADAM17, TNF-*α*, and sMER) were observed between the obese and nonobese groups, opening the opportunity to identify potential biomarkers for therapeutic exploration.

## 2. Materials and Methods

### 2.1. Participants

This was a cross-sectional study aimed to investigate the impact of central obesity on efferocytosis signaling in visceral adipose tissue. A total of 103 male participants were randomly selected to participate in this study, with recruitment conducted from January to August 2022. Participants were initially divided into two groups based on a predefined criteria for further analysis, with 56 participants with central obesity (obese group) and 29 nonobese individuals (control group). However, 18 participants were excluded from the study due to various reasons, including acute inflammation, impaired liver function, and a history of carcinoma. The initial division into the two groups aimed at exploring the potential differences between the control and overall obese groups. To further investigate potential variations in specific efferocytosis markers (PS, ADAM17, TNF-*α*, and sMER) within the obese population, the obese group was subsequently subdivided based on WC range into three subgroups (the level of obesity): obese 1 group (WC: 91–110 cm), obese 2 group (WC: 110–120 cm), and obese 3 group (WC > 120 cm).

Obesity was diagnosed according to WHO criteria for the Asia-Pacific region, which defines obesity as a body mass index (BMI) of ≥25 kg/m^2^ and central obesity as a WC ≥ 90 cm [3]. The number of participants in this study was calculated by using the formula for the quantitative variable with [19]

(1) $$\begin{array}{c} n=Z_{1-\alpha /2}^{2}\frac{\left(1-P\right)}{\varepsilon^{2}P}, \end{array}$$

where *n* is the number of samples, *P* is the proportion of subjects in the study at 0.5 with a confidence interval of 95%, *Z*_1−*α*/2_ is the expected significance at 1.96, *ε* is the estimated prevalence of obesity in Indonesia at around 21.8% based on the 2018 Indonesia Basic Health Research [20], and *n* is the minimum sample size of 81 subjects. Participants in this study were men 30–50 years old, with normal liver functions, and no evidence of acute infection, a history of autoimmune disorders, or malignancies during examination. The presence of autoimmune disorders or cancer among the participants was assessed using a questionnaire to ensure the validity of the study.

### 2.2. Ethical Approval

The study was carried out in accordance with the Declaration of Helsinki and the study protocol was approved by the Ethics Committee of Universitas Padjadjaran with approval number 983/UN6.KEP/EC/2021. Informed consent was obtained from each participant before the study started.

### 2.3. Clinical Evaluation

To ensure accurate preanalytical testing, a comprehensive questionnaire and medical history were used to record vital information about the participants. The questionnaire focused on various aspects, including fasting duration as a preanalytical requirement, medication use, parental disease history of the participant (such as cancer, autoimmune disorders, hypertension, diabetes, and cardiovascular disease), and lifestyle factors (such as smoking, physical activity, and alcohol consumption).

### 2.4. Anthropometric Measurement

Anthropometric measurements were performed to assess the weight in kilograms (kg) and height in centimeters (cm) of the participants to calculate their BMI. In addition, the WC was measured midway between the lowest rib and the top of the hipbone. Blood pressure (BP) was manually measured twice by using a sphygmomanometer while participants were in a relaxed seated position.

### 2.5. Biomarker Analysis

The present study used a variety of biomarker measurements to assess the metabolic and inflammatory status of the participants. Serum levels of glutamic-oxaloacetic transaminase (SGOT) and serum glutamic pyruvic transaminase (SGPT) were determined using the enzyme kinetics method, which followed the protocol of the International Federation of Clinical Chemistry, and measured using an Autoanalyzer ARCHITECT c4000 (Abbott, USA). The values obtained from these measurements were used to identify people with impaired liver function, where a normal SGOT : SGPT ratio is <1 [21]. Participants with impaired liver function were excluded from the study.

High-sensitivity C-reactive protein (hs-CRP) levels were measured using chemiluminescent method assay kits (Siemens, USA), which were centrifuged at 3000 rpm for 15 min prior to analysis. The level of hs-CRP was used to differentiate between participants with acute infection (hs-CRP >10 mg/L) and those without (hs-CRP <10 mg/L) [22]. The study excluded participants with acute infections to ensure that hs-CRP levels accurately reflected chronic inflammatory status.

The metabolic syndrome was identified using international criteria, including dyslipidemia and hyperglycemia. The diagnostic criteria for metabolic syndrome in men were based on the NCEP ATP III and the American Heart Association guidelines and included WC greater than 40 inches (>102 cm), BP greater than 130/85 mmHg, fasting triglyceride (TG) level >150 mg/dL, fasting HDL <40 mg/dL, and fasting blood sugar >100 mg/dL [23]. The American Diabetes Association recommends the use of HbA1c as an indicator of the risk of diabetes, as it has a less variability daily and is less affected by disease and stress. The HbA1c test was used to diagnose type 2 diabetes mellitus (T2DM) using HPLC, which is certified by the National Glycohemoglobin Standardization Program. The samples used were whole blood, and the HbA1c concentration was analyzed using HbA1c assay kits (Variant Turbo, Bio-Rad, USA) according to the manufacturer's instructions. The cholesterol profile was measured using an enzymatic colorimetric method in ARCHITECT c4000 (Abbott, USA), which included total cholesterol, low-density lipoprotein cholesterol (LDL cholesterol), high-density lipoprotein cholesterol (HDL cholesterol), and TG, to determine the presence of dyslipidemia. Dyslipidemia is diagnosed when one of the serum lipids is outside the normal reference range, where non-HDL cholesterol = total cholesterol − HDL cholesterol.

TNF-*α*, ADAM17, PS, and sMER levels were quantified using the quantitative sandwich enzyme-linked immunosorbent assay (ELISA) method on an ELISA analyzer. The samples were incubated for 30 min and centrifuged at 3000 rpm for 15 min before examination to determine the levels of each marker. These measurements were classified as research test groups and the test kits used included TNF-*α* (cat. no. HSTA00D, R&D System, USA), ADAM17 (cat. no. DY008, R&D System, USA), PS (cat. no. MBS2031953, MyBioSource, USA), and sMER (cat. no. ab119604, Abcam, USA).

### 2.6. Statistical Analysis

The results were presented as the mean ± standard error of mean (SEM) of the replicate assays, and the normality of the data was assessed prior to selecting the parametric tests. The Kolmogorov–Smirnov method was used to determine the distribution of the data (normal/abnormal) between all groups. The Mann–Whitney method was used to compare the two groups, and the correlation between two quantitative parameters was evaluated using the Spearman correlation coefficient. IBM SPSS version 23.0 statistical software (IBM, USA) was utilized for all statistical analyses and graph creation. An acceptable 5% error margin was established, and the confidence interval was established at 95%. Therefore, a *p* value of <0.05 was considered significant.

## 3. Results

### 3.1. Characteristics of the Participant

A total of 103 male participants aged 30–50 years were randomly selected to participate in this study. Participants were divided into two groups, the obese group and the nonobese (control) group, according to eligibility requirements for BMI and WC. Eighteen participants were excluded from the study. Twelve were excluded from the obese group due to acute inflammation, while six participants showed potentially impaired liver function (5 in the obese group and 1 in the control group). Furthermore, a participant in the obese group had a history of hepatocellular carcinoma. Finally, 85 individuals were retained for further analysis, with 56 participants in the obese group and 29 in the control group.

The demographic and metabolic characteristics of the participants are presented in Table 1. BMI and WC were significantly higher in the obese group (*p* < 0.001), with values of 34.1 ± 0.6 kg/m^2^ and 115.9 ± 1.2 cm than 22.7 ± 0.4 kg/m^2^ and 84.1 ± 0.9 cm in the control group, respectively. These results confirmed that the participants were selected according to the study inclusion criteria. There was no age difference between the groups (39.3 ± 0.7 vs. 38.5 ± 0.9 years in the obese and control groups, respectively). Data showed that higher BMI and WC were associated with higher rates of comorbidity. Mean levels of serum SGOT and SGPT were significantly higher in the obese group than in the control group (*p* < 0.05), while the SGOT : SGPT ratio was unremarkable (<1). Furthermore, the obese group had a significantly higher prevalence of diabetes (25% vs. 0%) and prediabetes (35% vs. 7%) than the control group, respectively, with a mean HbA1c value of 6.2% ± 0.2% compared to 5.3% ± 0.6% in the control group (*p* < 0.001). In terms of the classification of cardiovascular risk based on hs-CRP level [24], half of the participants in the obese group had a level of >3.0 mg/L, indicating a higher cardiovascular risk compared to only 14% in the control group. hs-CRP levels in the obese and control groups were statistically different (*p* < 0.001), with values of 5.2 ± 0.4 and 1.6 ± 0.4 mg/L, respectively. We found that 98% and 52% of participants in the obese and control groups, respectively, had dyslipidemia. Serum lipid levels, including LDL cholesterol, HDL cholesterol, and TG, were significantly different in the obese group compared to the control group (*p* < 0.05). Furthermore, we found that 67% of participants in the obese group and 7% in the control group had hypertension. Systolic and diastolic BP levels in the obese group were significantly higher than those in the control group (*p* < 0.001), with values of 140.6 ± 2.2 vs. 124.2 ± 2.2 mmHg and 94.5 ± 1.5 vs. 79.9 ± 1.6 mmHg, respectively.

Based on the classification of metabolic syndrome, 41 of 65 obesity participants (63%) met the criteria for metabolic syndrome, while no participants in the control group had metabolic syndrome. These results suggest an association between obesity and various metabolic and cardiovascular risk factors.

### 3.2. Correlation of WC with Metabolic Syndrome

In this study, the correlation between WC and components of metabolic syndrome was explored, including BMI, SGOT, SGPT, HbA1c, hs-CRP, and cholesterol profiles. As presented in Table 2, a significant positive association was observed between WC and BMI (*p* < 0.001). Given this finding, we considered the subsequent correlations with the WC levels. WC was found to be significantly positively correlated with SGOT and SGPT (*p* < 0.05) and the relationship of WC with HbA1c and hs-CRP also showed a strong positive correlation (*p* < 0.001), indicating that increased WC may be a marker of poor glycemic control and chronic inflammation. A strong positive correlation was observed between WC and LDL cholesterol, TG, and non-HDL cholesterol (*p* < 0.05). In particular, a robust negative correlation was found between WC and HDL cholesterol (*p* < 0.001). Furthermore, WC exhibited a significant positive correlation with BP levels (*p* < 0.001), implying that individuals with a larger WC may have a greater risk of hypertension.

### 3.3. Efferocytosis in the Obese and Control Groups

The efferocytosis process in this study was observed by measuring the levels of PS, ADAM17, TNF-*α*, and sMER as shown in Table 3. The mean level of PS in the obese group was higher than that in the control group; however, this difference was not statistically significant. Similarly, mean levels of TNF-*α* and sMER were higher in the obese group than in the control group, although the difference was not statistically significant. Interestingly, the findings revealed that the ADAM17 level was significantly higher in the obese group than in the control group (*p* < 0.001).

Based on these observations, we proceeded to examine the levels of PS, ADAM17, TNF-*α*, and sMER based on the WC ranges. As presented in Table 4, PS levels differ significantly between the control group and the obese group 3 (*p* < 0.05), but no significant differences were observed between the control group and obese groups 1 and 2. Furthermore, there is a significant difference in PS levels between obese groups 2 and 3 (*p* < 0.05). Table 4 further illustrates that the mean PS levels in obese group 1 are lower than those in the control group. Furthermore, significant differences in sMER levels are evident between the control group and the obese group 3 (*p* < 0.05), as well as between the obese groups 2 and 3 (*p* < 0.05). Interestingly, a similar decrease pattern is observed for sMER levels in groups 1 and 2, which have lower levels than the control group.

Regarding ADAM17 levels, significant differences are observed from the control group to the obese group 1 (*p* < 0.05) and similarly between the control group and the obese groups 2 and 3. However, TNF-*α* levels show no significant differences between the control group and obese groups 1, 2, and 3 (*p* > 0.05). In Table 4, it is evident that PS, ADAM17, and sMER levels differ significantly between the control group and the obese group 3 that had WC > 120 cm.

### 3.4. Correlation of PS, ADAM17, TNF-*α*, and sMER Levels

Table 5 provides a comprehensive overview of the correlation between variables. It reveals a statistically significant positive correlation (*p* = 0.001) between WC and PS, with correlation coefficients (*r*) of 0.354. Furthermore, Spearman's correlation coefficient between PS and ADAM17 was positive and statistically significant (*r* = 0.303, *p* < 0.05). The correlation between ADAM17 and TNF-α was also positive and statistically significant (*r* = 0.300, *p* < 0.05), suggests that ADAM17 may play a regulatory role in TNF-*α* levels. Furthermore, Table 5 shows a statistically significant positive correlation (*r* = 0.230, *p* < 0.05) between ADAM17 and sMER. However, it does not reveal a significant correlation between TNF-*α* and hs-CRP, as evidenced by the low correlation coefficient (*r* = 0.025) and the high *p* value (*p* > 0.05). A significant positive correlation (*r* = 0.548, *p* < 0.001) was observed between PS and TNF-*α* (*r* = 0.548, *p* < 0.001), further strengthening the correlation between PS-ADAM17 and ADAM17-TNF-*α*.

## 4. Discussion

Obesity has been shown to trigger cellular apoptosis and inflammation in adipose tissue, exacerbating the risk of various detrimental health outcomes, including but not limited to T2DM, dyslipidemia, hypertension, and hepatic steatosis. Collectively, these conditions constitute a syndrome referred to as “metabolic syndrome” [25, 26]. Intriguingly, our study revealed that more than 50% of participants with obesity were unaware of their status of metabolic syndrome. This finding validates previous investigations that reported a positive association between increased visceral adiposity and increased symptoms of metabolic syndrome symptoms [27, 28].

Adipose tissue is a major source of inflammatory markers in obesity. During the inflammatory process, visceral adipose tissue also produces proinflammatory cytokines and contributes to an increase in serum hs-CRP levels by increasing its signaling [29, 30]. Previous studies have revealed a strong positive association between obesity measurements, such as WC and hs-CRP [31]. Our research also found a strong positive correlation between WC and hs-CRP levels (*p* < 0.001). Hs-CRP is made by the liver in response to inflammatory cytokines such as TNF-*α*. Unfortunately, a significant correlation between hs-CRP and TNF-*α* was not detected in this study. According to Table 3, there were no statistically significant differences in the TNF-*α* levels between the obese and control groups. Although there was an increasing trend in the mean value of the obese group, the average value remained relatively constant. The lack of a significant correlation between hs-CRP and TNF-*α* in this research could be due to several factors. One possibility is that the history of other infections such as COVID-19 could influence this result, particularly in the control group, where 59% of the participants had previously been infected with COVID (compared to 48% in the obese group) and had been disease-free for at least three months before participating in this research. TNF-*α* levels were significantly higher in patients with postacute COVID-19 than in those with no prior exposure to COVID-19, according to a German cohort study that recruited post-COVID-19 patients [32]. Elevated levels of TNF-*α* remained stable in individuals with ongoing postacute COVID-19 sequelae who experienced prolonged symptoms until 10 months after COVID-19 testing [32]. Even though the hs-CRP value was established at <10 mg/L to demonstrate that there was no acute infection, it appears that cellular inflammation occurs post-COVID-19. However, as a chronic inflammatory marker, hs-CRP in the obese group differed significantly from the control group (*p* < 0.001), which indicates a higher chronic inflammation in the obese group. Another possibility is that other inflammatory markers or signaling pathways may promote the observed association between obesity and hs-CRP levels, independently of TNF-*α*. For example, adipose tissue can produce a variety of other proinflammatory cytokines, such as IL-6, that can contribute to the observed increase in hs-CRP levels in obese individuals. Other studies have suggested that factors such as oxidative stress and insulin resistance may play a role in the link between obesity and inflammation [33].

Inflammation, metabolic processes, and cell death are highly interrelated processes in obesity. One of the most obvious signs of cell death is the movement of PS from the inside to the outside of the leaflet of the cell plasma membrane. A previous study by Solá et al. [34] compared the expression of PS in obese patients and a control group and it was reported that obese patients had a significantly higher PS expression than the control group. However, another previous study by Samocha-Bonet et al. did not find any difference in the expression of PS between the two groups, although the expression of PS showed an increasing trend in the obese group [35]. In our study, we found that the mean level of PS was higher in the obese group than in the control group, and this increase was correlated with the increase in WC (*p* < 0.05). The discrepancy between the results of this study and Solá et al. does not appear to be related to the sample size (49 cases); however, it could be related to the different degrees of obesity, since the mean BMI levels and WC in the study by Sola et al. were 46.1 ± 6.5 kg/m^2^ and 127 ± 15 cm, respectively [34]. The further analysis we performed is similar to the study by Solá et al. [34], which indicates that in the obese group with WC greater than 120 cm in this study, the levels of PS are significantly different from those in the control group. Based on these findings, the degree of obesity appears to contribute to the elevation of PS levels.

Externalization of PS has traditionally been considered as an “eat-me” signal for apoptotic cells, where PS is transferred to the outer leaflet of the cell membrane, leading to ADAM17 activation [36, 37]. In this study, a significant positive correlation was observed between PS and ADAM17 levels (*p* < 0.05). Although no statistically significant differences were found in PS levels between the obese and control groups, our findings indicated higher PS levels in the obese group than in the control group. Furthermore, PS levels were higher in the obese group with WC greater than 120 cm than in the control group. In this study, ADAM17 levels differed significantly between the obese and control groups, even between the control group and the obese group with a WC of 91–110 cm. In addition to PS, other factors involved in ADAM17 regulation in obesity should also be considered, as previous research has shown that obesity is characterized by a deficiency of TIMP3 in white adipose tissue and liver, resulting in elevated ADAM17 levels [38, 39]. Furthermore, alterations in ERK or p38 MAPK signaling pathways have been shown to affect the dynamic balance between the conformations of ADAM17 dimers and monomers [38].

ADAM17 plays a crucial role in the proteolytic release of proinflammatory cytokines from cellular membranes, with TNF-*α* being a major cytokine processed by ADAM17, produced by various cell types and contributes to inflammation [7]. This study revealed a strong positive correlation between ADAM17 and TNF-*α* (*p* < 0.05). Furthermore, this study revealed a significant disparity in ADAM17 levels between the obese and control groups, suggesting that ADAM17 plays a multifaceted role beyond its involvement in the production of proinflammatory cytokines. Previous studies have shown that ADAM17 regulates more than 90 substrates that are involved in various cellular processes, including inflammation signaling, immune response, phagocyte infiltration, and cell development [40, 41].

Our findings also revealed a strong positive correlation (*p* < 0.001) between PS and TNF-*α* levels, which is consistent with the established correlations between PS and ADAM17, and ADAM17 and TNF-*α*. This suggests that the regulation of TNF-*α* may be influenced by PS through its association with ADAM17. Although TNF-*α* levels did not show a statistically significant difference between the obese and control groups, a significant difference was observed in ADAM17 levels between the two groups (*p* < 0.001). Unfortunately, we did not measure other cytokines that are regulated by ADAM17, such as IL-6 and CX3CL-1, preventing a comparison of their levels with TNF-*α*. We acknowledge that the measurement of markers in obesity may depend on the degree of obesity that can influence cellular processes at different stages, which could potentially explain the lack of significant differences in markers such as TNF-*α* and PS, as ongoing cellular repair processes may be occurring. We also suggest that at a certain degree of obesity, especially the mechanism of hyperplasia plays a significant role in compensating for the hypertrophic effect, which results in a potential anti-inflammatory effect that mitigates the proinflammatory consequences. This suggests that cellular processes in obesity are complex and multifaceted, with a delicate balance between proinflammatory and anti-inflammatory effects.

In obesity, adipose cell hypertrophy is a possible risk factor for cellular damage and death [42]. To comprehend the process that leads to cell death, we examined its correlation with inflammatory markers such as TNF-*α* and its regulator, ADAM17. As part of the cell remodeling process, macrophages play a vital role in maintaining tissue homeostasis by engaging in efferocytosis and preventing secondary necrosis [36]. When macrophages infiltrate adipose tissue, they arrange themselves around dead cells and form a CLS to engulf and eliminate the dead cells [43]. Moreover, the CLS also secretes proinflammatory factors such as TNF-*α* [44]. During efferocytosis, the macrophage MERTK functions as crucial receptors in the binding process [14], and in this study, we focused on measuring sMER levels. Although there were no statistically significant differences in sMER levels between the obese and control groups, the mean level of sMER in the obese group was higher than that in the control group. This finding differs from that of Suresh Babu et al. [16], who reported that *in vitro* efferocytosis was impaired in macrophages from obese and diabetic mice due to increased levels of sMER, leading to a decline in MERTK function. In addition, in this study, it was revealed that sMER levels were significantly different between the control group and the obese group with a WC greater than 120 cm. Interestingly, our study suggests that the degree of obesity appears to regulate changes in sMER levels, similar to what we observed with PS levels. However, our study revealed a positive correlation between sMER and ADAM17 (*p* < 0.05).

The positive correlation between sMER and ADAM17 is consistent with the findings of Babu et al., who reported that disruption of efferocytosis occurs through the release of MERTK by ADAM17, which acts as the main protease, and produces sMER [16]. However, research on sMER in humans using serum samples remains limited, and more studies with larger sample sizes are required to confirm these findings. To further explore the correlation between sMER and cellular function, additional research is needed to compare sMER levels obtained through *in vitro* and serum samples. Such a study would be valuable, since sMER circulates in the bloodstream and investigating its correlation with cellular function could provide insight into the mechanisms underlying the potential risk of cellular damage and death in central obesity.

**Figure 1:**
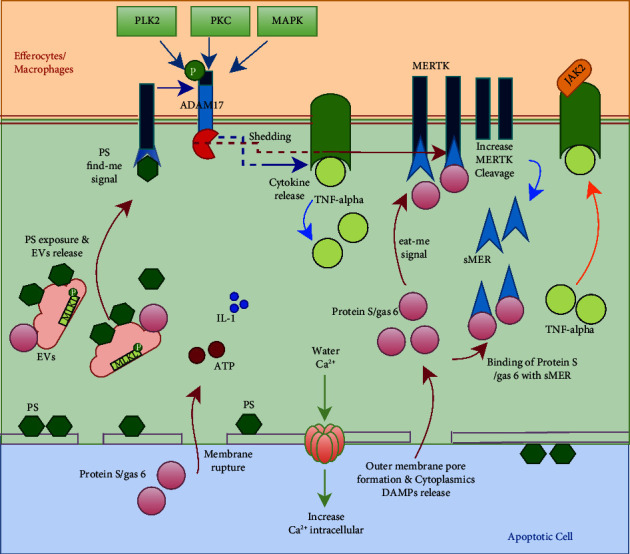
Illustrating the mechanism of efferocytosis impairment in central obesity. The cellular mechanism in central obesity continually leads to the damage and death of adipocytes. This is shown by the movement of PS to the outside of the cell membrane and the release of protein S/Gas6 and extracellular vesicles (EVs). Phagocytic cells, primarily macrophages, recognize PS as a “find me” signal. Meanwhile, protein S/Gas6 acts as an “eat-me” signal, binding to MERTK on phagocytic cells to initiate the clearance of damaged or dead cells and their components. PS, once bound to its receptor in phagocytic cells, activates ADAM17, which cleaves various cytokines such as TNF-*α*, serving as an inflammation marker. This leads to an increase in circulating TNF-*α*. However, ADAM17 also cleaves MERTK in phagocytic cells, generating sMER in the circulation. When sMER is high, it interferes with efferocytosis because it binds to the protein S/Gas6, which is made by damaged or dead cells. Consequently, competition arises between MERTK and sMER, which interferes with the efferocytosis process.

Based on the results and the correlation between the parameters mentioned above, we illustrate the efferocytosis process in central obesity in Figure 1.

## 5. Conclusions

Our research investigated the relationship between obesity and inflammatory markers, cell death, metabolic syndrome, and efferocytosis. Identified markers may be valuable in the development of effective therapies. The results demonstrated that central obesity triggered inflammation and cell death in adipose tissue, increasing the risk of metabolic syndrome. Impaired clearance of dead cells and debris in central obesity led to their accumulation, which activated immune cells and induced cytokine regulators such as ADAM17 to increase the inflammatory milieu. This cycle of cell death, impaired clearance, and inflammation further disrupted efferocytosis with increased levels of sMER. Thus, we conclude that PS, ADAM17, and sMER play an important role in efferocytosis activity in central obesity. More research is required to fully elucidate the intricate relationship between obesity, inflammation, cellular death cells, efferocytosis, and metabolic processes to develop improved preventive and treatment therapy.

## Figures and Tables

**Table 1:** Clinical characteristics of the participants.

Variables	Group		*p* value
	Control (*n* = 29)	Obese (*n* = 56)	
Age (years old)	38.5 ± 0.9	39.3 ± 0.7	0.197
BMI (kg/m^2^)	22.7 ± 0.4	34.1 ± 0.6	<0.001^*∗*^
WC (cm)	84.1 ± 0.9	115.9 ± 1.4	<0.001^*∗*^
SGOT (U/L)	22.2 ± 1.3	30.2 ± 2.2	0.006^*∗*^
SGPT (U/L)	28.7 ± 5.1	52.1 ± 4.2	<0.001^*∗*^
HbA1c (%)	5.3 ± 0.6	6.2 ± 0.2	<0.001^*∗*^
hs-CRP (mg/L)	1.6 ± 0.4	5.2 ± 0.4	<0.001^*∗*^
Total cholesterol (mg/dL)	200.4 ± 6.3	215.9 ± 4.8	0.050^*∗*^
LDL cholesterol (mg/dL)	121.1 ± 5.7	153.5 ± 4.5	<0.001^*∗*^
HDL cholesterol (mg/dL)	51.5 ± 1.8	41.0 ± 0.9	<0.001^*∗*^
Triglycerides (mg/dL)	105.3 ± 8.1	163.3 ± 9.0	<0.001^*∗*^
Non-HDL cholesterol (mg/dL)	148.9 ± 6.0	174.9 ± 4.8	0.001^*∗*^
BP systolic (mmHg)	124.2 ± 2.2	140.6 ± 2.2	<0.001^*∗*^
BP diastolic (mmHg)	79.9 ± 1.6	94.5 ± 1.5	<0.001^*∗*^

All results are expressed as mean ± SEM. BMI, body mass index; WC, waist circumference; SGOT, serum glutamic-oxaloacetic transaminase; SGPT, serum glutamic pyruvic transaminase; HbA1c, hemoglobin A1c; hs-CRP, high-sensitivity C-reactive protein; LDL, low-density lipoprotein; HDL, high-density lipoprotein; BP, blood pressure. ^*∗*^Significant in 95% confidence interval using the Mann–Whitney test.

**Table 2:** Correlation of WC with routine biomarker results and BP levels in the study group.

Variables	WC (cm)	
	Correlation coefficient (*r*)	*p* value
BMI (kg/m^2^)	0.935	<0.001^*∗*^
SGOT (U/L)	0.206	0.044^*∗*^
SGPT (U/L)	0.435	<0.001^*∗*^
HbA1c (%)	0.423	<0.001^*∗*^
hs-CRP (mg/L)	0.500	<0.001^*∗*^
Total cholesterol (mg/dL)	0.143	0.166
LDL cholesterol (mg/dL)	0.346	0.001^*∗*^
HDL cholesterol (mg/dL)	−0.514	<0.001^*∗*^
Triglycerides (mg/dL)	0.482	<0.001^*∗*^
Non-HDL cholesterol (mg/dL)	0.286	0.005^*∗*^
Systolic (mmHg)	0.462	<0.001^*∗*^
Diastolic (mmHg)	0.524	<0.001^*∗*^

^*∗*^Significant in the 95% confidence interval using Spearman's correlation test. BP, blood pressure.

**Table 3:** Comparison of PS, ADAM17, TNF-*α*, and sMER in obese vs. control groups.

Variables	Group		*p* value
	Control (*n* = 29)	Obese (*n* = 56)	
PS (ng/mL)	153.2 ± 15.6	190.5 ± 14.9	0.463
ADAM17 (pg/mL)	143.2 ± 59.8	1133.0 ± 238.5	<0.001^*∗*^
TNF-*α* (pg/mL)	1.2 ± 0.1	1.3 ± 0.7	0.584
sMER (pg/mL)	546.2 ± 163.0	595.1 ± 85.2	0.283

All results are expressed as mean ± SEM. PS, phosphatidylserine; ADAM17, a disintegrin and metalloprotease domain 17; TNF-*α*, tumor necrosis factor alpha; sMER, soluble MER. ^*∗*^Significant in the 95% confidence interval using the Mann–Whitney test.

**Table 4:** Comparison of PS, ADAM17, TNF-*α*, and sMER based on WC ranges.

Variables	Group				a	b	c	d	e
	Control (*n* = 29)	Obese 1 (WC 91–110 cm) (*n* = 17)	Obese 2 (WC 110–120 cm) (*n* = 22)	Obese 3 (WC > 120 cm) (*n* = 17)					
PS (ng/mL)	153.2 ± 15.6	135.4 ± 18.0	170.4 ± 16.9	285.0 ± 34.9	0.240	0.749	0.003^*∗*^	0.147	0.014^*∗*^
ADAM17 (kg/m^2^)	143.2 ± 59.8	400.9 ± 142.3	643.1 ± 198.7	2700.1 ± 679.4	0.011^*∗*^	<0.001^*∗*^	<0.001^*∗*^	0.404	0.015^*∗*^
TNF-*α* (pg/mL)	1.2 ± 0.1	1.2 ± 0.9	1.1 ± 0.8	1.6 ± 0.2	0.216	0.159	0.083	0.863	0.009^*∗*^
sMER (pg/mL)	546.2 ± 163.1	514.6 ± 82.9	406.3 ± 45.2	967.6 ± 272.8	0.825	0.825	0.026^*∗*^	0.343	0.007^*∗*^

All results are expressed as mean ± SEM. Obese 1, waist circumference 91–110 cm; obese 2, waist circumference 110–120 cm; obese 3, waist circumference >120 cm; ^a^comparison of confidence interval between the control group and obese 1 group; ^b^comparison of confidence interval between the control group and obese 2 group; ^c^comparison of confidence interval between the control group and obese 3 group; ^d^comparison of confidence interval between the obese 1 and obese 2 groups; ^e^comparison of confidence interval between the obese 2 and obese 3 groups. ^*∗*^Significant in the 95% confidence interval (*p* < 0.05) using the Mann–Whitney test.

**Table 5:** Correlation between each parameter in the study group.

Variables		WC (cm)	hs-CRP (mg/L)	PS (ng/mL)	ADAM17 (pg/mL)	TNF-*α* (pg/mL)	sMER (pg/mL)
WC (cm)	*r*	—	0.500	0.354	0.554	0.141	0.246
	*p*	—	<0.001^*∗*^	0.001^*∗*^	<0.001^*∗*^	0.205	0.025^*∗*^

hs-CRP (mg/L)	*r*	0.500	—	0.201	0.297	0.025	0.104
	*p*	<0.001^*∗*^	—	0.069	0.007^*∗*^	0.820	0.351

PS (ng/mL)	*r*	0.354	0.201	—	0.303	0.548	0.332
	*p*	0.001^*∗*^	0.069	—	0.006^*∗*^	<0.001^*∗*^	0.002^*∗*^

ADAM17 (pg/mL)	*r*	0.554	0.297	0.303	—	0.300	0.230
	*p*	<0.001^*∗*^	0.007^*∗*^	0.006^*∗*^	—	0.006^*∗*^	0.038^*∗*^

TNF-*α* (pg/mL)	*r*	0.141	0.025	0.548	0.300	—	0.473
	*p*	0.205	0.820	<0.001^*∗*^	0.006^*∗*^	—	<0.001^*∗*^

sMER (pg/mL)	*r*	0.246	0.104	0.332	0.230	0.473	—
	*p*	0.025^*∗*^	0.351	0.002^*∗*^	0.038^*∗*^	<0.001^*∗*^	—

^*∗*^Significant in the 95% confidence interval using the Spearman correlation test.

## Data Availability

All data used in this study are available upon request from the corresponding author.
